# O Paradoxo da Intensidade do Exercício Físico na Prevenção de Eventos Cardiovasculares na Doença Arterial Obstrutiva Periférica

**DOI:** 10.36660/abc.20210595

**Published:** 2021-08-09

**Affiliations:** 1 Clínica Cardiosport FlorianópolisSC Brasil Clínica Cardiosport, Florianópolis SC - Brasil.; 2 Instituto de Cardiologia de Santa Catarina São JoséSC Brasil Instituto de Cardiologia de Santa Catarina (ICSC), São José SC - Brasil.

**Keywords:** Doença da Artéria Coronariana, Reabilitação, Fatores de Risco, Claudicação Intermitente, Exercício, Caminhada, Atividade Motora

A doença arterial periférica (DAP), que tem como característica o estreitamento das artérias de membros inferiores por acometimento aterosclerótico, apresenta manifestações clínicas que vão muito além de apenas uma redução do fluxo sanguíneo, levando à isquemia crônica. Evidências atuais mostram que disfunção endotelial, estresse oxidativo, rigidez arterial e inflamação também acarretam o comprometimento funcional e, consequentemente, o declínio do paciente.^1^

Todos esses fatores acabam impactando na qualidade de vida do indivíduo, pois reduzem sua resistência ao caminhar, tendo a claudicação intermitente (CI) como principal sintoma. Não menos importante, também verificamos danos progressivos às fibras musculares ocasionados por essa isquemia crônica, corroborando ainda mais para a disfunção da morfologia musculoesquelética e metabólica do membro. Isso acaba criando uma barreira importante para a prática de atividade física, perpetuando e elevando os riscos de eventos cardiovasculares^1^^–^^3^

Para a redução desses fatores, as diretrizes consideram o exercício físico como ferramenta essencial na abordagem terapêutica. Estudos randomizados (RCT) demonstram que, apesar de não obtermos melhora do índice tornozelo- braquial (ITB) com essa abordagem, conseguimos ampliar o tempo de caminhada e a distância máxima percorrida (DMP) e neutralizar a CI; portanto, melhorar a qualidade de vida. Em 30 RCT, incluindo 1.816 pacientes com CI, a distância percorrida livre de dor e a DMP aumentaram em média 82 e 109m, respectivamente, em até dois anos.^4^^–^^9^

Em uma metanálise de 25 estudos randomizados (1.054 pacientes) abordando estratégias de exercícios em reabilitação de pacientes com DAP, foi concluído que o exercício em esteira supervisionado foi superior ao grupo-controle, com um ganho de 128m na distância de caminhada sem dor e de 180m na distância de caminhada máxima. Em contrapartida, 3 RCT (n=493) com DAP demonstraram que exercícios de caminhada domiciliares, quando incorporados com técnicas de mudança comportamental, melhoraram a distância de caminhada no teste de 6 minutos mais do que intervenções em esteira supervisionada (45-54m *vs*. 33-35m, respectivamente). Esse fato, talvez, possa ser em virtude da maior facilidade e aplicabilidade dos exercícios em solo em comparação à esteira, que requer tempo de aprendizagem.^5^^–^^8^

No entanto, além dos benefícios demonstrados, é importante compreender os riscos inerentes ao grau de intensidade do exercício físico neste grupo de paciente em questão, uma vez que, agudamente, cada sessão pode aumentar transitoriamente seu risco cardiovascular. Estudos anteriores relataram que caminhar com sintomas quase máximos de CI promove aumento de sobrecarga cardíaca, disfunção endotelial, estresse oxidativo e inflamação.^6^^–^^9^

Nesta edição dos Arquivos Brasileiros de Cardiologia, Marcel Chehuen et al. compararam, em pacientes com DAP sintomática, os efeitos fisiológicos agudos de exercícios máximos e submáximos de caminhada no pós-exercício. Dos 50 pacientes selecionados, apenas 30 foram incluídos no estudo. As variáveis analisadas foram: função cardiovascular, frequência cardíaca (FC) e sua variabilidade, modulação autonômica, função vascular e endotelial, estresse oxidativo e inflamação. Foi possível observar, quanto aos efeitos agudos, uma redução da pressão arterial (PA) sistólica após o teste submáximo, ao contrário da sessão máxima que aumentou com significância estatística. Já em relação à PA diastólica, houve aumento apenas com a caminhada máxima (p<0,001) assim como o duplo produto (p=0,007). As variáveis como FC e inflamação (ICAM e VCAM) tiveram aumentos semelhantes e com significância para ambos os testes. Quando foram analisadas as variáveis de estresse oxidativo e função endotelial, não houve alterações nos valores de óxido nítrico e capacidade vasodilatadora entre as sessões; portanto, sem significância estatística.^10^

De fato, a hipotensão pós-teste submáximo já havia sido relatada em estudos anteriores, mas não para o teste máximo, que, inclusive, até aumentou a pressão arterial nesses pacientes. Isso poderia implicar como terapia adicional no tratamento dos pacientes hipertensos e com DAP, utilizando a caminhada submáxima, mas não máxima, promovendo benefícios hipotensivos crônicos nessa população. Além disso, a sua prescrição estaria mais adequada do que a caminhada máxima, pois resultaria em um menor risco cardiovascular agudo durante o período de recuperação.

Vale salientar que, apesar de encontrarmos resultados importantes e interessantes neste estudo, ele foi realizado apenas com homens e em estágios de Fountain IIa/IIb, e não pode ser extrapolado para mulheres ou em outros estágios da doença, visto que as respostas fisiológicas poderiam ser diferentes. Destaca-se, ainda, ser um estudo unicêntrico, com um número pequeno de participantes incluídos, sendo ainda necessários estudos randomizados maiores, incluindo mulheres e outros estágios da doença para, assim, conseguir superar essas limitações.^10^
