# Supplementary material for: O Paradoxo da Intensidade do Exercício Físico na Prevenção de Eventos Cardiovasculares na Doença Arterial Obstrutiva Periférica
Source: Arq Bras Cardiol. 2021 Aug 9;117(2):317–8. [Article in Portuguese] doi: 10.36660/abc.20210595 (PMC8395792; doi:10.36660/abc.20210595)
Supplement: Translation

# Translation: The Paradox of Exercise Intensity in Preventing Cardiovascular Events in Peripheral Arterial Occlusive Disease

**Affiliations:** 1 FlorianópolisSC Brazil Clínica Cardiosport, Florianópolis SC - Brazil.; 2 São JoséSC Brazil Instituto de Cardiologia de Santa Catarina (ICSC), São José SC - Brazil.

**Keywords:** Coronary Artery Disease, Rehabilitation, Risk Factors, Intermittent Claudication, Exercise, Walking, Motor Activity

Peripheral arterial disease (PAD) is characterized by narrowing of the arteries of the lower limbs due to atherosclerotic involvement. Its clinical manifestations go far beyond just a reduction in blood flow, leading to chronic ischemia. Current evidence shows that endothelial dysfunction, oxidative stress, arterial stiffness and inflammation also lead to functional impairment, consequently to patient decline.^1^

All these factors end up impacting the quality of life of individuals, as it reduces their walking resistance, with intermittent claudication (IC) as the main symptom. Last but not least, we have also found progressive damage to muscle fibers caused by this chronic ischemia, further worsening the dysfunction of the skeletal muscle and metabolic morphology of the limb. This ends up creating an important barrier to the practice of physical activity, perpetuating and increasing the risk of cardiovascular events.^1^^–^^3^

To reduce these factors, the guidelines consider physical exercise as an essential tool in the therapeutic approach. Randomized controlled trials (RCT) demonstrate that although we did not obtain an improvement in the ankle-brachial index (ABI) with this approach, we were able to extend walking time, the maximum walking distance (MWD), neutralize IC, and therefore improve the quality of life. In 30 RCTs, including 1816 patients with IC, pain-free walking distance and MWD increased on average 82 and 109 meters, respectively, in up to two years.^4^^–^^9^

A meta-analysis of 25 randomized studies (1,054 patients) addressing exercise strategies in the rehabilitation of patients with PAD, found that supervised treadmill exercise was better than the control group, with a gain of 128 meters in pain-free walking distance and 180 meters at the maximum walking distance. In contrast, 3 RCTs (n=493) with PAD found that home walking exercises, when combined with behavior change techniques, improved the 6-minute walking test distance more than interventions on a supervised treadmill (45–54 meters vs. 33–35 meters, respectively). This fact, perhaps, may be due to the greater ease and applicability of exercises on the ground compared to the treadmill, which requires learning time.^5^^–^^8^

However, in addition to the benefits shown, it is also important to understand the risks inherent to the degree of intensity of physical exercise in this group of patients, since each session may acutely increase their cardiovascular risk temporarily. Previous studies have reported that walking with near-maximal IC symptoms promotes increased cardiac overload, endothelial dysfunction, oxidative stress, and inflammation.^6^^–^^9^

In this issue of Arquivos Brasileiros de Cardiologia, Marcel Chehuen et al. compared the acute physiological effects of post-exercise maximal and submaximal walking exercises in patients with symptomatic PAD. Of the 50 selected patients, only 30 were included in the study. The variables analyzed were: cardiovascular function, heart rate (HR) and its variability, autonomic modulation, vascular and endothelial function, oxidative stress and inflammation. It was possible to observe, regarding the acute effects, a reduction in systolic BP after the submaximal test, as opposed to the maximum session, which increased with statistical significance. Regarding diastolic BP, there was an increase only with the maximum walk (p<0.001) as well as the double product (p=0.007). Variables such as HR and inflammation (ICAM and VCAM) had similar increases with statistical significance for both tests. And when the variables of oxidative stress and endothelial function were analyzed, there were no changes in the values of nitric oxide and vasodilator capacity between the sessions, therefore without statistical significance.^10^

In fact, submaximal post-test hypotension had been reported in previous studies, but not for the maximal test, which even increased blood pressure in these patients. This could imply an additional therapy for hypertensive and PAD patients, using submaximal — not maximal — walking exercise to promote chronic hypotensive benefits in this population. Besides, its prescription would be more appropriate than maximal walking exercise, as it would result in a lower acute cardiovascular risk during the recovery period.

Although we found important and interesting results in this study, it only included men, in Fountain IIa/IIb stages, and cannot be extrapolated to women or to any other stages of the disease, as physiological responses could be different. Also worthy of note, this is a single-center study including a small number of participants. We still need larger randomized studies, including women and other stages of the disease, in order to overcome these limitations.^10^
